# Tachykinins Stimulate a Subset of Mouse Taste Cells

**DOI:** 10.1371/journal.pone.0031697

**Published:** 2012-02-21

**Authors:** Jeff Grant

**Affiliations:** Department of Physiology and Biophysics, University of Miami School of Medicine, Miami, Florida, United States of America; German Institute for Human Nutrition, Germany

## Abstract

The tachykinins substance P (SP) and neurokinin A (NKA) are present in nociceptive sensory fibers expressing transient receptor potential cation channel, subfamily V, member 1 (TRPV1). These fibers are found extensively in and around the taste buds of several species. Tachykinins are released from nociceptive fibers by irritants such as capsaicin, the active compound found in chili peppers commonly associated with the sensation of spiciness. Using real-time Ca^2+^-imaging on isolated taste cells, it was observed that SP induces Ca^2+^ -responses in a subset of taste cells at concentrations in the low nanomolar range. These responses were reversibly inhibited by blocking the SP receptor NK-1R. NKA also induced Ca^2+^-responses in a subset of taste cells, but only at concentrations in the high nanomolar range. These responses were only partially inhibited by blocking the NKA receptor NK-2R, and were also inhibited by blocking NK-1R indicating that NKA is only active in taste cells at concentrations that activate both receptors. In addition, it was determined that tachykinin signaling in taste cells requires Ca^2+^-release from endoplasmic reticulum stores. RT-PCR analysis further confirmed that mouse taste buds express NK-1R and NK-2R. Using Ca^2+^-imaging and single cell RT-PCR, it was determined that the majority of tachykinin-responsive taste cells were Type I (Glial-like) and umami-responsive Type II (Receptor) cells. Importantly, stimulating NK-1R had an additive effect on Ca^2+^ responses evoked by umami stimuli in Type II (Receptor) cells. This data indicates that tachykinin release from nociceptive sensory fibers in and around taste buds may enhance umami and other taste modalities, providing a possible mechanism for the increased palatability of spicy foods.

## Introduction

Spices that contain capsaicin, such as chili powder, are commonly used to increase the palatability of food in certain cultures. Capsaicin, as well as high temperature, activates the transient receptor potential cation channel, subfamily V, member 1 (TRPV1), found on a subpopulation of sensory afferent nociceptive nerve fibers [1]. TRPV1 is a receptor for painful heat sensation, which explains why capsaicin produces a burning sensation [2]. However, it is not clear as to why a pungent compound such as capsaicin is commonly used and enjoyed in the foods of many cultures.

Substance P (SP) and neurokinin A (NKA) are excitatory peptides of the tachykinin family. They are found extensively in capsaicin-sensitive peripheral sensory fibers [3]. A third member of the tachykinin family, Neurokinin B, is generally not expressed in peripheral sensory fibers [4]. In response to activation of TRPV1 by capsaicin and other painful stimuli, sensory nerve fibers release SP and NKA at their peripheral terminals [5]. Release of these tachykinins from peripheral fibers modulates gastrointestinal motility [6], genitourinary tract function [7], immune responses [8], and many other physiological processes [9].

Nerve fibers containing SP and NKA are present in and around taste buds of several species [10], [11]. Several studies have shown that SP can directly stimulate or modulate physiological responses in gustatory neurons of the rostral nucleus tractus solitarius [12]–[14] and gustatory sensory ganglion [15]. In addition, intraventricular injections of the neurokinin 3 receptor (NK-3R) agonist senktide decreased salt intake in rats [16]. Wang *et al.* (1995) previously hypothesized that release of peptides such as SP from peripheral nociceptive fibers may modulate taste responses at the level of taste buds. Indeed, they demonstrated that direct stimulation of the lingual nerve, which projects the SP-containing fibers to the tongue, modulated responses of the chorda tymphani to salt solution [17]. In a separate study, the same group found the SP receptor neurokinin 1 receptor (NK-1R) immunohistochemically localized in taste cells of the rat [18]. However, to date no physiological studies to have been performed to determine if tachykinins can directly stimulate taste cells.

In this study, it is shown that the tachykinin receptors NK-1R and to a lesser extent the NKA selective neurokinin 2 receptor (NK-2R) are expressed in mouse taste buds. Activation of these receptors induced Ca^2+^-responses in taste cells. NK-1R had a much larger role these Ca^2+^-responses as compared to NK-2R. In addition, NK-1R-mediated Ca^2+^ responses were due to release of Ca^2+^ from intracellular stores. The majority of tachykinin- responsive taste cells were identified to be Type I (Glial-like) and umami-responsive Type II (Receptor) cells. In addition, activation of NK-1R had an additive effect on Ca^2+^ responses to umami stimulus in taste Type II (Receptor) cells, suggesting that tachykinins may enhance the taste sensation of umami and other taste modalities.

## Materials and Methods

### Animals

All experimental procedures were approved by the University of Miami Animal Care and Use Committee. C57BL/6J adult mice, as well as transgenic mice expressing enhanced green fluorescent protein (GFP) under control of the PLCβ2 promoter (PLCβ2–GFP mice) [19], and transgenic mice expressing GFP under the control of the GAD67 promoter (GAD67-GFP mice) [20] were euthanized by exposure to 100% CO_2_ until clinical death was achieved. Cervical dislocation was performed, and tongues were excised for further dissection.

### Isolated taste buds and taste cells

The lingual epithelium containing vallate mouse papillae was removed from the tongue by injecting an enzyme mixture (1 mg ml^−1^ collagenase A (Roche, Indianapolis, IN), 2.5 mg ml^−1^ dispase II (Roche, Indianapolis, IN), 0.25 mg ml^−1^ Elastase (Worthington, Lakewood, NJ), and 0.5 mg ml^−1^ DNAse I (Sigma, St. Louis, MO)) directly under the epithelium surrounding the taste papillae. The peeled epithelium was re-incubated for 2 min in the above mentioned enzyme mixture, then for 5 min in Ca^2+^/Mg^2+^-free Tyrode solution. Taste buds were gently drawn into fire-polished micropipettes with suction, and either processed for RNA extraction or transferred to a glass coverslip for isolated taste cell preparation. For isolated taste cell preparations, taste buds were incubated for 10 min in 0.25% trypsin, then triturated 20 times with a fire-polished micropipette and transferred the isolated cells to a shallow recording chamber with a glass coverslip coated with Cell-Tak (BD Biosciences, San Jose, California). Isolated taste cells were then loaded with 5 µM fura-2 AM for 45 min. Taste cells were perfused with Tyrode solution (in mM: 140 NaCl, 5 KCl, 2 CaCl_2_, 1 MgCl_2_, 10 HEPES, 10 glucose, 10 sodium pyruvate, 5 NaHCO_3_, pH 7.2–7.4, 310–320 mosmol/l). For experiments in nominal extracellular Ca^2+^, MgCl_2_ was substituted equimolar for CaCl_2_.

### RNA preparation and RT-PCR

RNA was isolated from isolated whole taste buds, isolated taste cells, and from pieces of non-taste lingual epithelium (enzymatically peeled). RNA was also isolated from mouse intestine and eye for positive controls. Total RNA was isolated using the Absolutely RNA nanoprep kit (Agilent Technologies, Santa Clara, CA). Any remaining DNA was eliminated with DNAse I digestion, and RNA was reverse-transcribed using Superscript III reverse transcriptase (Invitrogen, Carlsbad, CA). One to two taste bud equivalents of cDNA were used for subsequent whole-taste bud PCR reactions. Table 1 lists the sequences and annealing temperatures of primers used in this study. For the whole-taste bud studies, amplification was for 30 cycles (β-actin), 35 cycles (PLC-β2), or 40 cycles (NK-1R, NK-2R, NK-3R). For single-cell RT-PCR, amplification was for 45 cycles for all primers used. RT-PCR was performed on an iCycler (Biorad, Hercules, CA). PCR products were run on a 2% agarose gel and examined it under UV light using a gel imager (Cell Biosciences, Inc., Santa Clara, CA.).

**Table 1:** RT-PCR Primer sequences.

Target	Gene name	Accession #	Primer 1 (5′→3′)	Primer 2 (3′→5′)	Product length (bp)	Annealing temp.,°C
NK-1R	Tacr1	NM_009313.5	tcaatgacaggttccgtctg	ggtcttcgagttgcttcgag	260	60
NK-2R	Tacr2	NM_009314.4	caccatgtacaaccccatca	gcaccgtcttgcttcttttc	388	60
NK-3R	Tacr3	NM_021382.6	gtggtgacatttgccatctg	acaccagcgaaatgctctct	210	60
PLC-β2	Plcb2	NM_177568	ctcgctttgggaagtttgc	gcattgactgtcatcgggt	226	58
SNAP-25	Snap25	NM_011428	ggcaataatcaggatggagtag	agatttaaccacttcccagca	310	58
NTPDase2	Entptd2	NM_009849	agctggaggatgccacagag	gagagcaacccaggagctga	299	63
β-Actin	Actb	NM_007393	caccctgtgctgctcacc	gcacgatttccctctcag	328	58

### Ca^2+^ imaging

Isolated taste cells loaded with Fura-2 were viewed on Olympus Optical IX70 inverted microscope (Tokyo, Japan). Sequential fluorescent images were recorded at 10–20× magnification at a rate of 1 capture every 2 seconds using a band pass emission filter (510±80 nm) and with sequential excitation at 340 nm followed by 380 nm (F340/F380). Images were processed with Imaging Workbench v5 software (INDEC Biosystems, Mountain View, CA). F340/F380 ratios were converted to Ca^2+^ concentration values using a Fura-2 calcium calibration buffer kit (Invitrogen, Carlsbad, California) as follows:
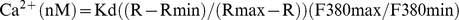
with Kd = 224 nM [21], R = measured ratio (F340/F380), R_min_ = ratio at zero free Ca^2+^, R_max_ = ratio at saturating Ca^2+^ (39 µM), F380_max_ is the fluorescence intensity at 380 nm, in zero free Ca^2+^ and F380_min_ is the fluorescence intensity at 380 nm, in saturating free Ca^2+^.

Measurable calcium responses were defined as an increase in [Ca^2+^] that was greater than 2 times that of the baseline Ca^2+^ fluctuation. Baseline [Ca^2+^] and baseline Ca^2+^ fluctuations (standard deviation) of the cells were calculated from 20–60 seconds of captures prior to stimulus application. The taste cell preparations had an average baseline [Ca^2+^] of 117 nM+/−5.3 nM SE (n = 234 cells). The average baseline Ca^2+^ fluctuation of the cells was 5.3 nM+/−0.8 nM SE (n = 234 cells).

### Stimulation

Chemicals were purchased from Sigma (St. Louis, MO) unless otherwise indicated. Isolated taste cells were stimulated by bath-perfusion of KCl (50 mM, substituted equimolar for NaCl), bitter taste mix (10 µM cyclohexamide, 1 mM denatonium), sweet taste mix (1 mM sucralose, 0.1 mM SC45647), monosodium glutamate (substituted equimolar for NaCl), glutamate+0.5 mM IMP, 2.5 mM IMP, substance P (SP, Tocris, Ellisville, Missouri), [Sar^9^,Met(O_2_)^11^]-Substance P (SSP, Tocris), neurokinin A (NKA, Tocris), [Lys^5^,MeLeu^9^,Nle^10^]-NKA(4–10) (L-NKA, Tocris), and senktide, (Tocris). All of the above chemicals were dissolved in Tyrode solution and stimuli were applied for 30 seconds followed by a rinse for several minutes.

### Identification of taste cell types

Type III (Presynaptic) cells were identified by Ca^2+^-influx when cells were exposed to 50 mM KCl. Type III cells have been previously been shown to express voltage-gated Ca^2+^ channels and respond well to KCl [22]. In contrast, Type II (Receptor) cells respond to specific taste qualities but not KCl depolarization [23]. Type II (Receptor) cells were categorized by their responsiveness to bitter (1 mM denatonium+10 µM cyclohexamide), sweet (1 mM sucralose+0.1 mM SC45647), or umami (30 mM glutamate+0.5 mM IMP or 2.5 mM IMP alone) stimuli. In addition to the umami taste receptors (T1R1/T1R3), several studies have demonstrated the likely presence of both ionotropic and metabotropic synaptic glutamate receptors on taste cells, sensitive to glutamate ≤1 mM [24]–[30]. Given this, taste cells were classified as umami-responsive Type II (Receptor) cells only if they demonstrated synergistic responses between glutamate and IMP, as 5′-ribonucleotides such as IMP strongly potentiate glutamate-induced responses from the umami receptor T1R1/T1R3 [31]. To independently identify Type II (Receptor) or Type III (Presynaptic) cells, isolated taste cells were used from transgenic mice expressing PLCβ2-GFP, a marker for Type II (Receptor) cells [19], and transgenic mice expressing GAD67-GFP, a marker for Type III (Presynaptic) cells [23]. For identification of cell types in single-cell RT-PCR experiments, expression of NTPdase II defined Type I (Glial-like) cells [32], expression of PLC-β2 defined Type II (Receptor) taste cells [33], and expression of SNAP-25 defined Type III (Presynaptic) taste cells [34].

### Cell counting

For determination of the responsiveness of all isolated cells in a dissociated taste bud preparation to tachykinins, tachykinin-responding cells were compared to all fura-2 loaded cells in a particular field. For determination of the overlap of tachykinin responsiveness with the various taste qualities and taste cell types, tachykinin responding cells were compared only to taste cells that could be identified physiologically, i.e. bitter, sweet and umami Type II (Receptor) cells and Type III (Presynaptic) cells, or taste cells identified by GFP fluorescence in the case of experiments performed using PLCβ2-GFP and GAD67-GFP mice. All counts were from at least three independent Ca^2+^ imaging experiments.

## Results

### Tachykinin receptors are expressed in mouse taste cells

**Figure 1:**
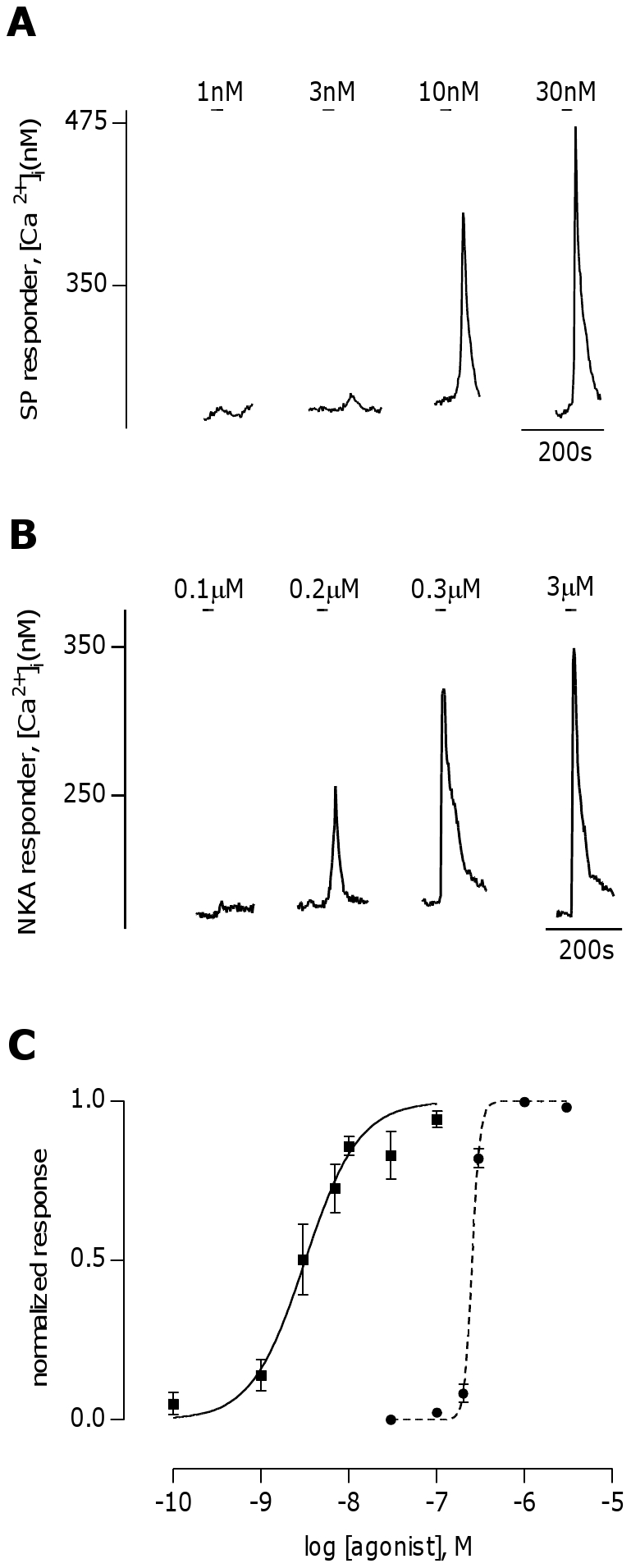
Substance P and Neurokinin A induce Ca^2+^-responses in mouse taste cells. **A**, Representative Ca^2+^ trace from a substance P (SP) responsive taste cell stimulated with 1,3, 10, and 30 nM SP. **B**, Representative Ca^2+^ trace from a neurokinin A (NKA) responsive taste cell stimulated with 0.1 ,0.2, 0.3, and 3 µM NKA. **C**, Dose response curves for SP (solid line, n = 33 cells) and NKA (dotted line, n = 37 cells) in isolated taste cells, normalized to the concentration of agonist that gives the highest Ca^2+^-response in a given cell. SP had an EC_50_ of 3.2 nM, while NKA had an EC_50_ of 256 nM.

A previous immunohistochemical study has demonstrated that rat taste cells express the tachykinin receptor NK-1R [18]. To determine if activation of tachykinin receptors present on mouse taste cells can cause physiological responses, functional imaging was conducted on isolated cells from dissociated taste bud preparations obtained from C57BL/6J mice, loaded with the Ca^2+^-sensitive dye fura-2. All three subtypes of tachykinin receptors have previously been shown to induce IP_3_ formation through G-protein- coupled signaling [35]. As such, the presence of functional tachykinin receptors on taste cells would be indicated by Ca^2+^-release from intracellular stores in response to specific agonists to these receptors. Substance P (SP), bath-applied at concentrations in the low nanomolar range (1–30 nM), induced Ca^2+^-responses in a subset of the fura-2 loaded cells in the preparation (∼9%, 54/627 cells, Fig. 1A). It was also observed that Neurokinin A (NKA), which preferentially activates NK-2R, also induced Ca^2+^-responses in a similar number of fura-2 loaded cells, although only at concentrations above 100 nM (∼9%, 54/605 cells, Fig. 1B). The NK-3R agonist senktide did not induce Ca^2+^-responses in cells at concentrations up to 3 µM (data not shown), indicating that NK-3R is not present in mouse circumvallate taste buds. Figure 1C shows dose-response relationships for SP and NKA, with SP having an EC_50_ of 3.2 nM (n = 33) and NKA having an EC_50_ of 256 nM (n = 37).

**Figure 2:**
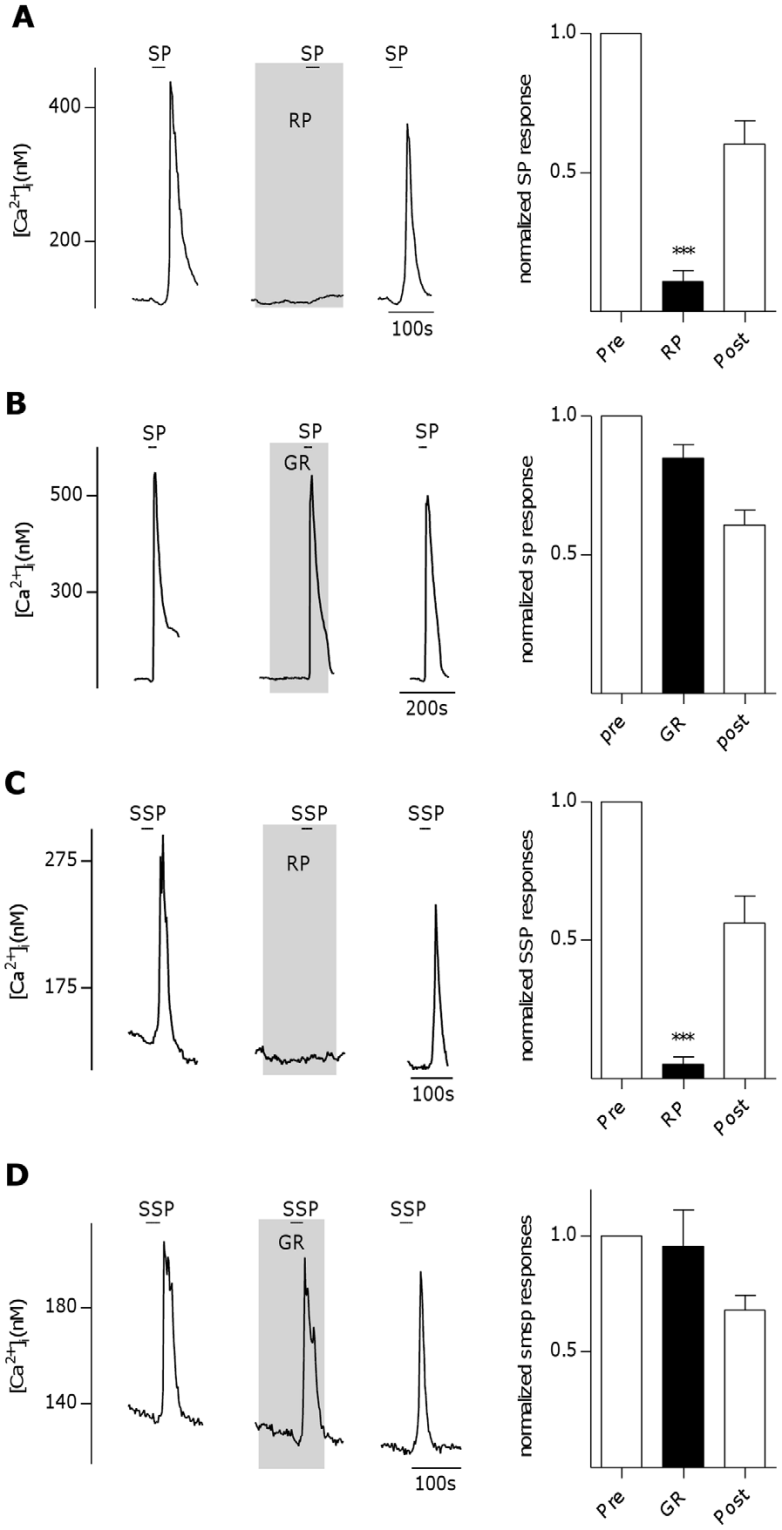
Substance P-induced Ca^2+^-responses in mouse taste cells involve activation of the neurokinin 1 receptor. **A**, Inhibition of Ca^2+^- responses to substance P (SP, 10 nM) in an isolated taste cell by the neurokinin 1 receptor (NK-1R) antagonist RP67580 (RP, 100 nM). Right panel: RP67580 reversibly inhibits SP-induced Ca^2+^-responses. Bars represent averaged normalized peak responses from 9 cells (***P<0.0001, repeated measures ANOVA). **B**, Example trace from isolated taste cell showing no inhibition of Ca^2+^-responses to SP (10 nM) by the neurokinin 2 receptor (NK-2R) antagonist GR159897 (GR,1 µM). Right panel: GR159897 does not inhibit SP-induced Ca^2+^- responses. Bars represent averaged normalized peak responses from 9 cells. **C**, Inhibition of Ca^2+^- responses to NK-1R-selective agonist [Sar^9^,Met(O_2_)^11^]-Substance P (SSP, 3 nM) in an isolated taste cell by RP67580 (RP, 30 nM). Right panel: RP67580 reversibly inhibits SSP-induced Ca^2+^-responses. Bars represent averaged normalized peak responses from 4 cells (***P<0.0001, repeated measures ANOVA). **D**, Example trace from isolated taste cell showing no inhibition of Ca^2+^-responses to SSP (3 nM) by GR159897 (1 µM). Right panel: GR159897 does not inhibit SSP-induced Ca^2+^ responses. Bars represent averaged normalized peak responses from 8 cells.

SP and NKA preferentially activate NK-1R and NK-2R, respectively. However, at sufficiently high concentrations, SP and NKA activate all three neurokinin receptors [36]. To determine if SP specifically acts on NK-1R in taste cells, NK-1R was blocked with the selective NK-1R antagonist RP67580 (100 nM, Tocris Bioscience, Ellisville, Mo.). Ca^2+^-responses elicited by SP (10 nM) were significantly and reversibly reduced by RP67580 (Fig. 2A, P<0.0001, repeated measures ANOVA, n = 9 cells). In this and many of the other experiments of this study, recovery responses after the washout of the antagonist often were smaller than the magnitude of the pre-antagonist responses. This could be due to several factors, such as desensitization of receptors, lingering antagonist effects, or declining health of the cells. In contrast, blocking NK-2R with selective antagonist GR159897 (1 µM Tocris Bioscience, Ellisville, Mo) had no effect on SP-induced Ca^2+-^responses (Fig. 2B, n = 9 cells). To further confirm the presence of NK-1Rs, taste cells were stimulated with [Sar^9^,Met(O_2_)^11^]-Substance P (SSP), a highly- selective agonist for NK-1R [37]. SSP induced Ca^2+^-responses in taste cells at concentrations similar to SP (EC_50_ = 3.41 nM). Like SP, Ca^2+^-responses induced by SSP (3 nM) were blocked by RP67580 (30 nM, Fig. 2C, P<0.0001, repeated measures ANOVA, n = 4 cells), but not GR159897 (1 µM, Fig. 2D, n = 8 cells). This data convincingly shows that SP induces Ca^2+^-responses in taste cells via activation of NK-1R.

**Figure 3:**
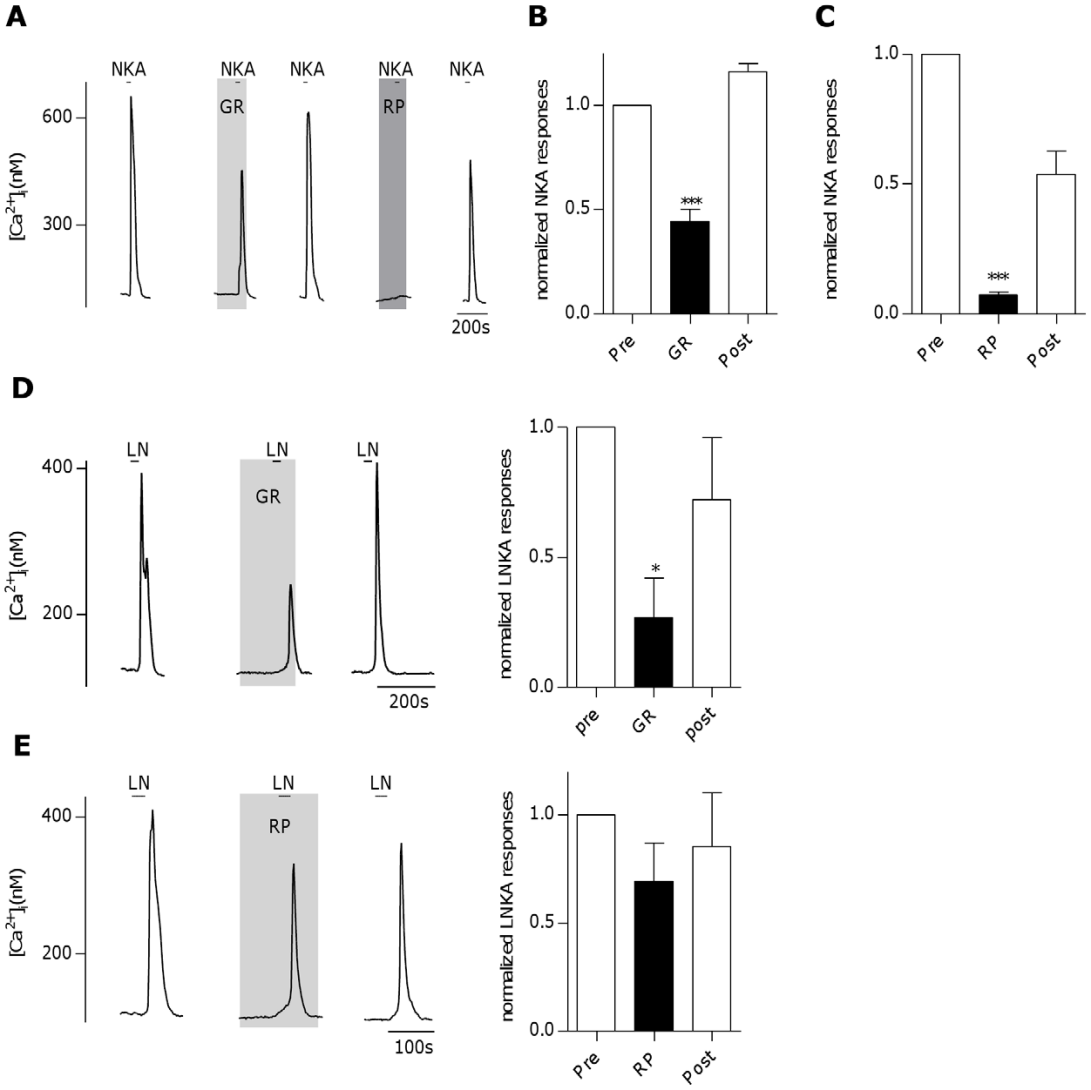
Neurokinin A-induced Ca^2+^-responses in mouse taste cells involve activation of both the neurokinin 1 and neurokinin 2 receptors. **A**, Inhibition of Ca^2+^- responses to neurokinin A (NKA, 300 nM) in an isolated taste cell by the neurokinin 2 receptor (NK-2R) antagonist GR159897 (GR,1 µM) and the neurokinin 1 receptor (NK-1R) antagonist RP67580 (RP, 1 µM). **B**, GR159897 partially and reversibly inhibits NKA-induced Ca^2+^- responses. Bars represent averaged normalized peak responses from 30 cells (***P<0.0001, repeated measures ANOVA). **C**, RP67580 reversibly inhibits NKA-induced Ca^2+^-responses. Bars represent averaged normalized peak responses from 7 cells (***P<0.0001, repeated measures ANOVA). **D**, Inhibition of Ca^2+^- responses to [Lys^5^,MeLeu^9^,Nle^10^]-NKA(4–10) (LN, 100 nM) in an isolated taste cell by GR159897 (GR, 300 nM). Right panel: GR159897 reversibly inhibits LN-induced Ca^2+^- responses. Bars represent averaged normalized peak responses from 7 cells (*P<0.05, repeated measures ANOVA). **E**, Example trace from isolated taste cell showing no significant inhibition of Ca^2+^-responses to LN (100 nM) by RP67580 (1 µM). Right panel: RP67580 does not inhibit LN-induced Ca^2+^- responses. Bars represent averaged normalized peak responses from 7 cells.

To test if NKA specifically activates NK-2Rs in taste cells, GR159897 was used to block NK-2Rs in taste cells stimulated with NKA (300 nM). NKA-induced Ca^2+^-responses were only partially blocked by GR159897 (1 µM Fig. 3A+B P<0.0001, repeated measures ANOVA, n = 30 cells), while almost fully blocked by RP67580 (1 µM) (Fig. 3A+C, P<0.0001, repeated measures ANOVA , n = 7 cells). Stimulating taste cells with [Lys^5^,MeLeu^9^,Nle^10^]-NKA(4–10) (L-NKA), a selective agonist for NK-2Rs resulted in Ca^2+^-responses only at concentrations at or above 100 nM. Responses induced by L-NKA (100 nM) were significantly but not completely blocked by GR159897 (300 nM, Fig. 3D, P<0.05, repeated measures ANOVA, n = 7 cells), while not significantly blocked by RP67580 (1 µM, Fig. 3E, n = 7 cells). However, there was a non-significant trend towards decreased magnitude of L-NKA responses with RP67580 treatment. These data suggest that L-NKA is likely not completely selective to NK-2R at concentrations at or above 100 nM. Therefore, while NK-2Rs are likely present on taste cells, NKA acts on taste cells only at concentrations that activate both NK-1R and NK-2Rs, suggesting relatively low levels of NK-2R expression. It also indicates that NK-1R and NK-2R are likely expressed in the same taste cells, with NK-2R playing a lesser role in tachykinin-induced responses as compared to NK-1R.

### NK-1R activation induces Ca^2+^ release from intracellular stores in taste cells

**Figure 4:**
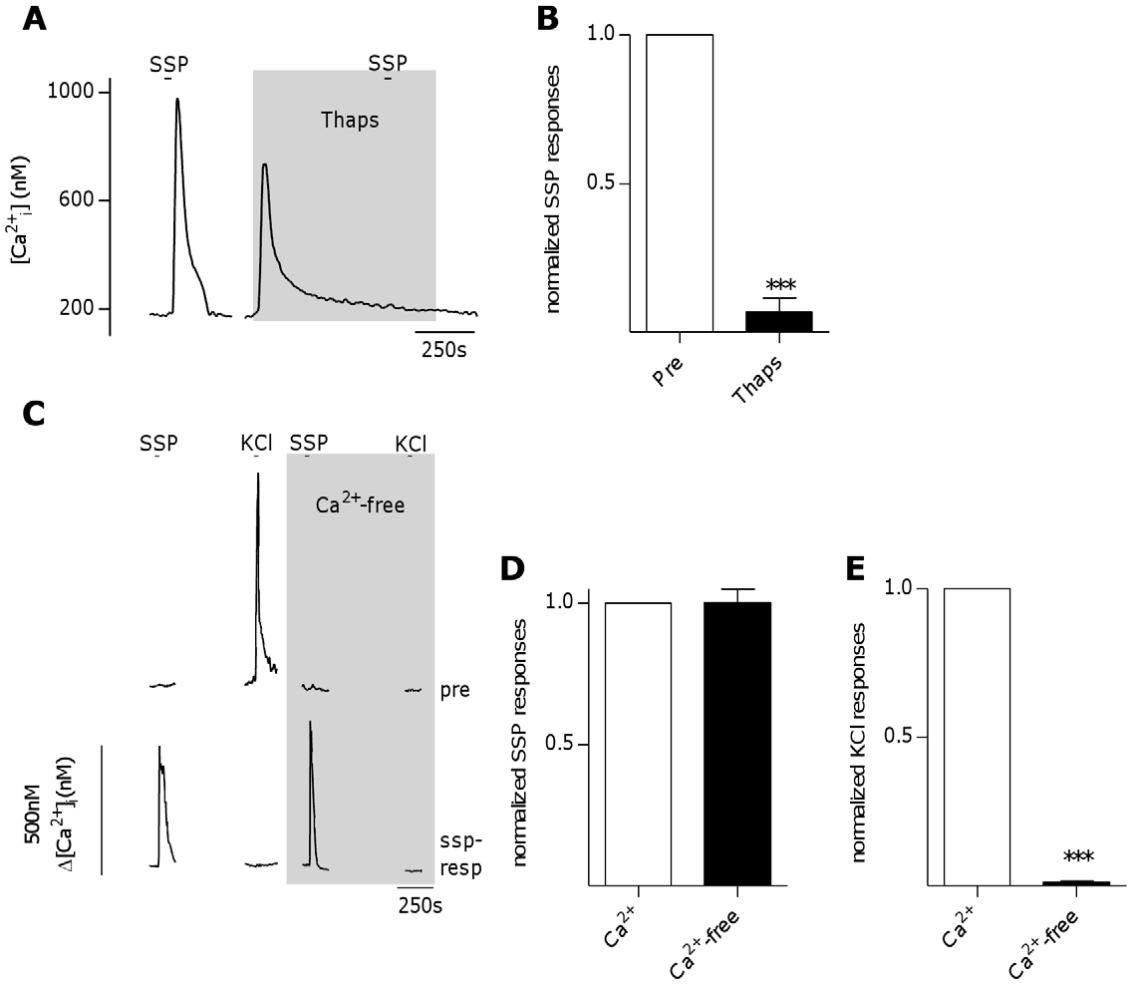
NK-1R activation induces Ca^2+^ release from intracellular stores in taste cells. **A**, Inhibition of Ca^2+^ responses to [Sar^9^,Met(O_2_)^11^]-Substance P (SSP, 10 nM) in an isolated taste cell after a 10 minute pre-treatment with the sarco-endoplasmic reticulum Ca^2+^-ATPase inhibitor thapsigargin (Thaps, 1 µM). **B**, Thapsigargin inhibits SSP-induced Ca^2+^-responses in taste cells. Bars represent averaged normalized peak responses from 10 cells (***P<0.0001, student's t-test). **C**, Representative Ca^2+^ traces from Type III (Presynaptic) and [Sar^9^,Met(O_2_)^11^]-Substance P (SSP)-responsive taste cells stimulated in both the presence and absence of extracellular Ca^2+^ in the same experimental run. The Type III (Presynaptic) cell (top trace) responded to 50 mM KCl only while extracellular Ca^2+^ was present, while the SSP-responsive cell (bottom trace) showed similar responses to 10 nM SSP in both the presence and absence of extracellular Ca^2+^. **D**, Ca^2+^ responses to 10 nM SSP in SSP-responsive taste cells were not significantly changed in the absence of extracellular Ca^2+^. Bars represent averaged normalized peak responses from 27 cells. **E**, Ca^2+^ responses to 50 mM KCl in Type III (Presynaptic) cells were eliminated in the absence of extracellular Ca^2+^. Bars represent averaged normalized peak responses from 11 cells (*** p<0.0001, Student's paired t-test).

It has been shown in several cell types that tachykinin receptors are G-protein-coupled receptors that induce Ca^2+^-release from intracellular stores in cells via phospholipase C -mediated IP_3_ production and subsequent activation of IP_3_ receptors on the endoplasmic reticulum [35]. To determine if tachykinin-induced Ca^2+^-signals in taste cells were in due to a similar pathway, NK-1R-expressing taste cells, identified by stimulation with 10 nM SSP, were treated for ∼10 min with the irreversible sarco-endoplasmic reticulum Ca^2+^-ATPase inhibitor thapsigargin (1 µM) in order to drain endoplasmic reticulum Ca^2+^-stores. SSP-induced Ca^2+^-responses were completely eliminated following thapsigargin treatment. (Fig. 4A+B, p<0.0001, student's paired t-test, n = 10 cells). To further confirm these results, taste cells were stimulated with SSP (10 nM) in both the presence and absence of extracellular Ca^2+^. The magnitude of SSP-induced Ca^2+^-signals were nearly identical in both the presence and absence of extracellular Ca^2+^ (Figure 4C+D, p = 0.6601, Student's paired t test, n = 27 cells). As a control to insure that basal Ca^2+^-levels in nominal Ca^2+^ buffer were low enough to eliminate signals generated due to Ca^2+^-influx through the plasma membrane, Type III (Presynaptic) cells, present in the same taste cell preparations, were stimulated with 50 mM KCl. Depolarization of Type III (Presynaptic) cells with KCl induces Ca^2+^-influx through voltage-gated Ca^2+^ channels [22]. Ca^2+^-signals induced by 50 mM KCl in Type III (Presynaptic) cells were completely eliminated in nominal Ca^2^ buffer (4C+E, p<0.0001, Student's paired t test, n = 11 cells), demonstrating that Ca^2+^-influx through the plasma membrane was absent. Thus, the Ca^2+^-signals induced by SSP in Ca^2+^-free buffer could only be due to release from the intracellular stores of taste cells. This confirms that tachykinin-mediated Ca^2+^-signaling in taste cells requires Ca^2+^ release from intracellular stores, but not influx of Ca^2+^ from the extracellular space.

### NK-1Rs are expressed in Type II (Receptor) cells

Given that NK-1R appears to be the main tachykinin receptor in taste cells, experiments were performed to identify which taste cells express NK-1R. There are three primary mature taste cell types in taste buds: Type II (Receptor) cells, Type III (Presynaptic) cells, and Type I (Glial-like) cells [38]. There is currently no physiological method to identify Type I (Glial-like) cells, so I initially focused on whether NK-1Rs are expressed in Type II (Receptor) cells and/or Type III (Presynaptic) cells.

**Figure 5:**
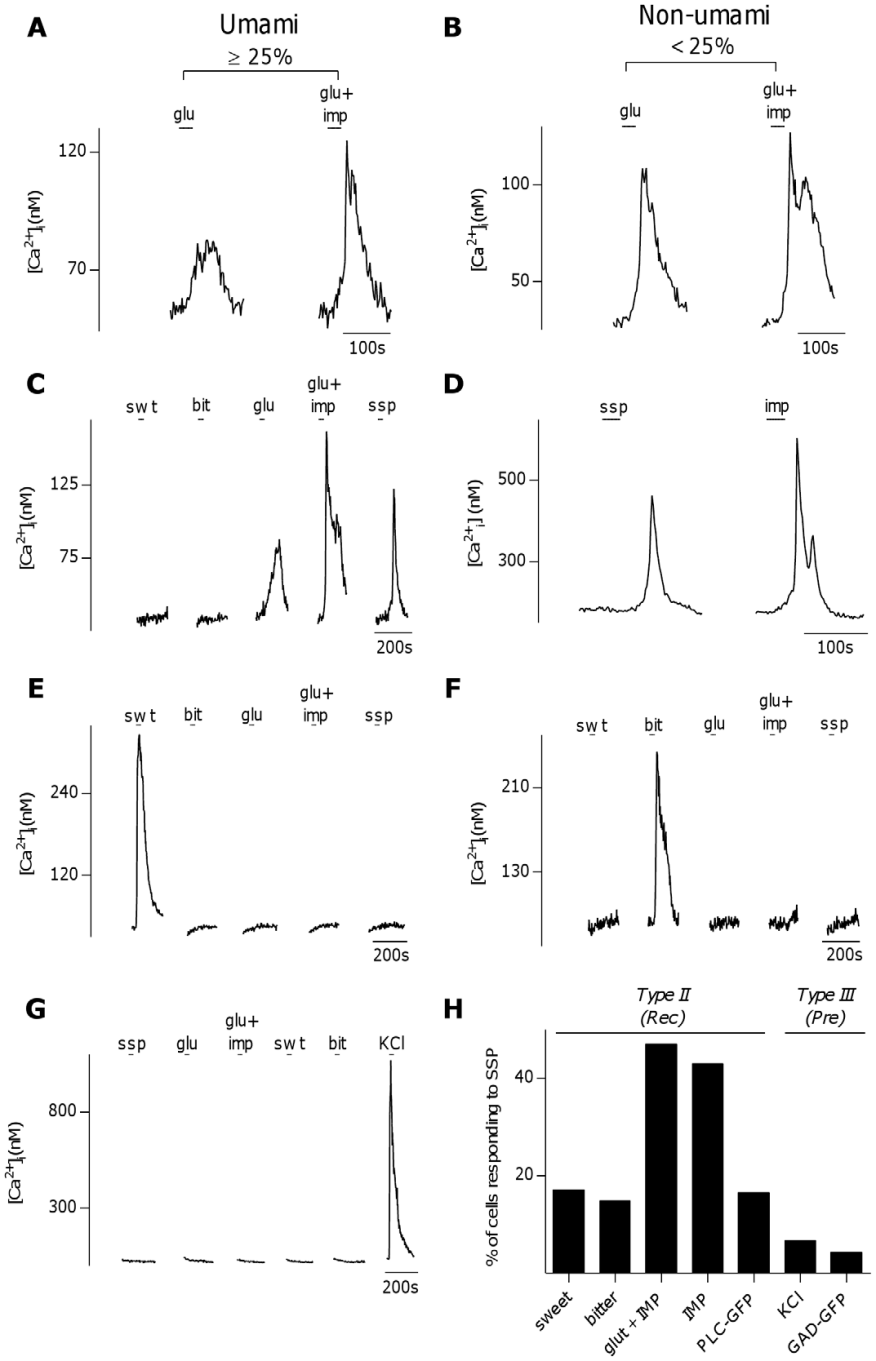
Activation of neurokinin 1 receptors induces Ca^2+^ responses in a subset of Type II (Receptor) cells. **A**, **B**, Physiological criteria for identifying umami Type II (Receptor) cells. **A**, Ca^2+^-responses to glutamate (30 mM) and glutamate+IMP (0.5 mM) in a taste cell. This cell showed a greater than 25% increase in peak response to glutamate in the presence of IMP, thus was defined as an umami cell. **B**. Another taste cell, which did not show a greater than 25% increase in peak response to glutamate in the presence of IMP. This cell was defined as a non-umami cell. **C**, An umami cell, characterized by increased sensitivity to glutamate (glu, 30 mM) in the presence of IMP (0.5 mM), also showed a Ca^2+^-response to [Sar^9^,Met(O_2_)^11^]-Substance P (ssp ,10 nM). A large number of umami cells (47%, 124/264 cells) responded to ssp. **D**, Similarly, sample trace of an umami cell, characterized by responsiveness to a high concentration of IMP alone (2.5 mM), which also responded to ssp (10 nM). **E+F**, sweet- (swt,**E**) or bitter- (bit, **F**) responsive cell that was unresponsive to bath-applied ssp, (10 nM). Only 6 of 35 (17.1%) sweet and 24 of 162 (14.8%) bitter taste cells responded to SSP. **G**, A Type III (Presynaptic) cell, characterized by Ca^2+^-influx through voltage gated calcium channels due to depolarization by KCl (50 mM), that was unresponsive to SSP (10 nM). Only 7 of 104 (6.7%) of physiologically identified Type III (Presynaptic) cells responded to SSP. **H**, Summary of the percent of physiologically identified Type II (Receptor) (Rec) and Type III (Presynaptic)(Pre) cells that responded to SSP.

Type II (Receptor) cells, characterized by their sensitivity to sweet, bitter, or umami taste stimulation, were tested for their sensitivity to bath-applied SSP (10 nM), which induced Ca^2+^ responses in the fura-2 loaded cells of the taste cell preparations at a proportion similar to SP (∼9%, 81/898 cells). Cells were categorized as umami-sensitive if 30 mM glutamate in the presence of 0.5 mM IMP evoked responses ≥25% larger than responses from 30 mM glutamate alone (Figure 5 A+B). Remarkably, umami-responsive cells were the most responsive to SSP. Nearly half the umami-sensitive cells responded to SSP (124/264 cells, Fig. 5C). To further confirm the identification of umami sensitive Type II (Receptor) cells, in a second series of experiments cells were stimulated with IMP alone (2.5 mM). 5′-ribonucleotides alone elicit umami sensation but do not stimulate synaptic glutamate receptors [39]. IMP-sensitive taste cells also responded to SSP at a similarly high incidence (136/316 cells, Fig. 5D, p = 0.6874, Fisher's exact test). In contrast, compared to umami cells, significantly fewer sweet-sensitive Type II (Receptor) cells responded to SSP (6/35, Fig. 5E p = 0.0194, Fisher's exact test), as was also the case for bitter-sensitive Type II (Receptor) cells (24/162, Fig. 5F p<0.0001, Fisher's exact test). Further, only a low incidence of Type III (Presynaptic) cells identified by responses to KCl depolarization responded to SSP as compared to umami Type II (Receptor) cells (7/104 cells, Fig. 5G, p<0.0001, Fisher's exact test). As an independent confirmation, taste cells were isolated from transgenic mice expressing the Type II (Receptor) cell marker PLCβ2-GFP, and from transgenic mice expressing the Type III (Presynaptic) cell marker GAD67-GFP. A subset PLCβ2-GFP positive taste cells responded to 10 nM SSP (21/127 cells, trace not shown). However, only 2/46 (4.3%) GAD67-GFP positive cells showed responses to SSP (trace not shown), confirming what was observed in Type III (Presynaptic) cells identified by KCl depolarization. The percent response of each cell type to SSP is summarized in figure 5H.

**Figure 6:**
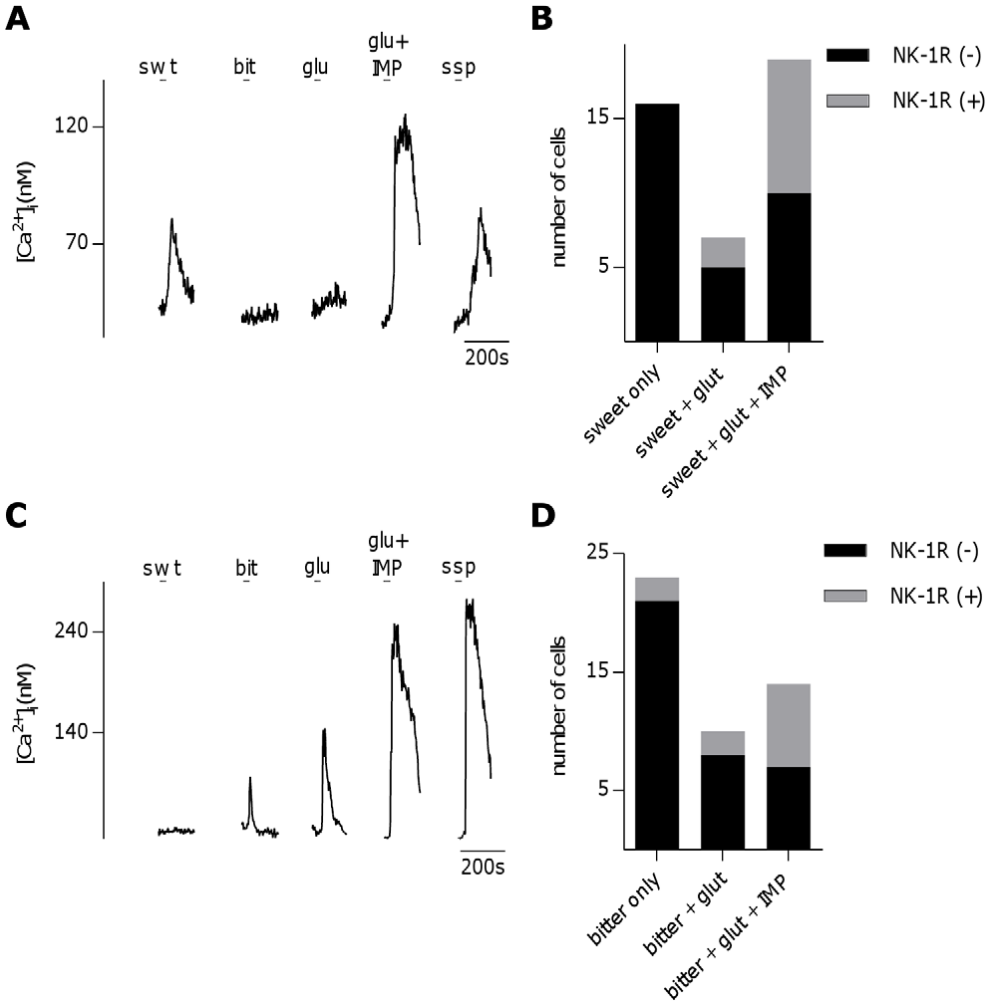
A subset of sweet and bitter Type II (Receptor) cells respond to umami stimuli and express neurokinin 1 receptors. **A**, Sweet- (swt) responsive taste cell that also showed Ca^2+^-responses to both umami stimuli (30 mM glu+0.5 mM IMP) and [Sar^9^,Met(O_2_)^11^]-Substance P (ssp,10 nM). **B**. Number of sweet only-, sweet- and glutamate-, and sweet-, glutamate- and IMP- responsive cells along with their respective expression of NK-1R as defined by responsiveness to ssp (10 nM). **C**, Bitter- (bit) responsive taste cell that also showed Ca^2+^-responses to both umami stimuli and SSP (10 nM). **D**, Number of bitter only-, bitter- and glutamate-, and bitter-, glutamate- and IMP- responsive cells along with their respective expression of NK-1R as defined by responsiveness to ssp (10 nM).

It should be noted that in the experiments and analysis above, I either separated the identification of NK-1R-expressing sweet, bitter, or umami Type II (Receptor) cells into independent experiments, or applied these three stimuli to the same groups of cells but only included Type II (Receptor) taste cells that were narrowly tuned to one taste modalility (namely sweet, bitter, or umami) in the subsequent analysis. However, in experiments where sweet, bitter, and umami stimuli were applied to the same group of cells, a large population of sweet or bitter responsive Type II (Receptor) cells were identified that were also responsive to 30 mM glutamate (26/42 sweet cells and 24/47 bitter cells), a subset of which showed enhanced glutamate response in the presence of 2.5 mM IMP (19/42 sweet cells and 14/47 bitter cells), indicating the presence of the umami T1R1-T1R3 receptor. These results were surprising as Type II (Receptor) cells are generally thought to be tuned to one taste modality (for review see [38]), although there is some conflicting evidence stating that that this may not be the case [40], [41]. Of sweet cells that were responsive to glutamate but showed no enhancement of glutamate response with IMP, 28.6% (2/7 cells, trace not shown) were also SSP responsive, while 47.4% (9/19) of sweet cells that showed IMP- enhanced glutamate responses were also SSP responsive (Fig. 6A). Of bitter cells that showed IMP-insensitive glutamate responses, 20% (2/10 cells, trace not shown) were SSP responsive, while of bitter cells that showed IMP-enhanced glutamate responses, 50% (7/14) were SSP responsive (Fig. 6C). These results are summarized in Fig. 6B and 6D for sweet and bitter cells, respectively. There were no observed Type II (Receptor) taste cells that responded to both sweet and bitter. This data suggests that expression of NK-1R is for the most part associated with Type II (Receptor) taste cells that express umami receptors, some of which may also express sweet or bitter receptors, while narrowly-tuned sweet or bitter Type II (Receptor) cells only rarely express NK-1R.

### NK-1R and NK-2R mRNA is detectable in taste buds by RT-PCR

**Figure 7:**
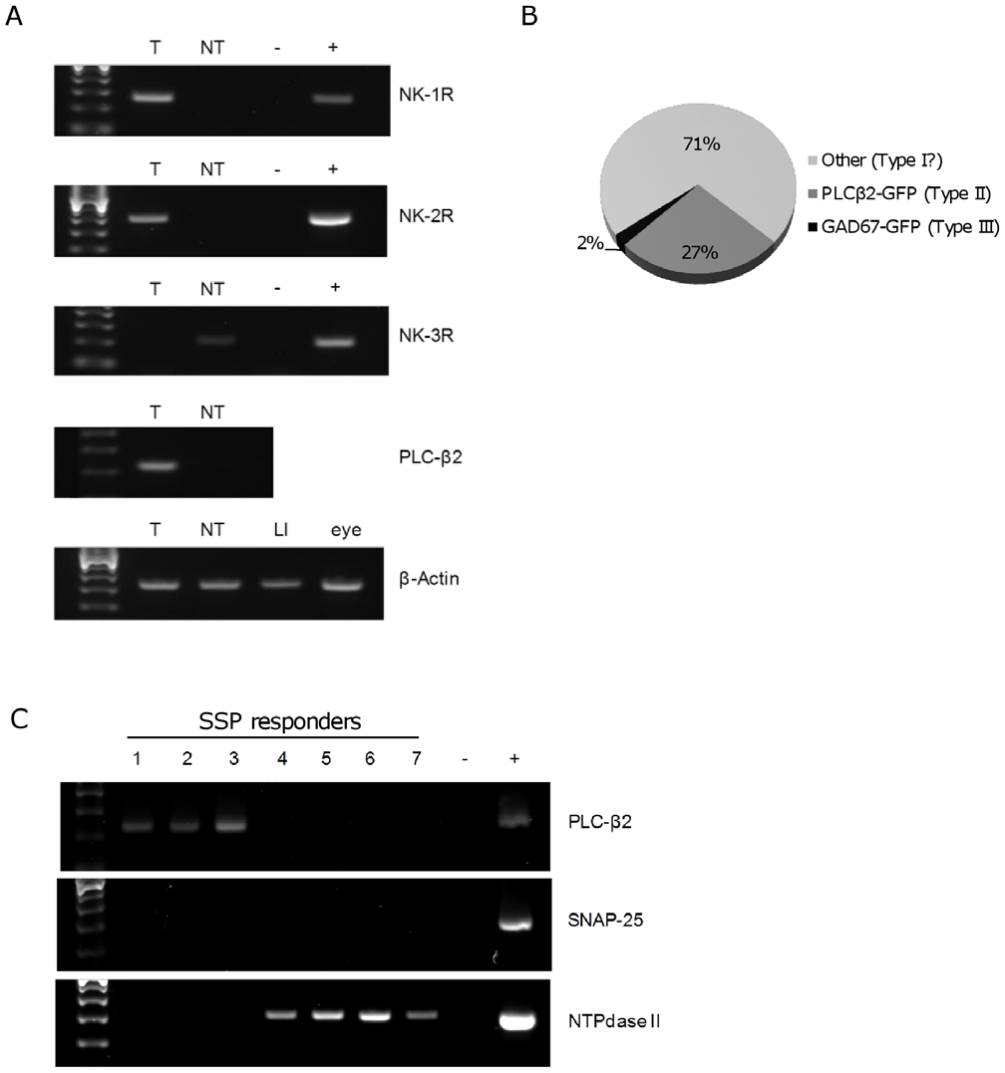
Neurokinin 1 receptor-expressing taste cells are Type I (Glial-like) cells and Type II (Receptor) cells. **A**, The tachykinin receptors NK-1R and NK-2R are detectable by RT-PCR in mouse taste buds. RT-PCR for the tachykinin receptors NK-1R, NK-2R, and NK-3R in isolated taste buds (T), non-taste epithelium (NT), a negative control lacking template (−), and a positive control tissue (+): Large intestine (LI) for NK-1R and NK-2R, eye for NK-3R. PLC-β2 was used to validate distinct taste bud cDNA versus non-taste epithelium cDNA, and β-Actin was a positive control for quality of the template cDNAs. **B**, Identity of cells responding to 10 nM [Sar^9^,Met(O_2_)^11^]-Substance P (SSP) in experiments using isolated taste cells from PLCβ2-GFP and GAD67-GFP transgenic mice. ∼27% of SSP responders were PLCβ2-GFP (+) (dark grey), while only ∼2% were GAD67-GFP (+) (black), indicating that a large proportion (∼70%) of SSP-responders were of an unknown cell type, possibly Type I taste cells (light grey). **C**, RT-PCR for PLC-β2, SNAP-25, and NTPdase II in isolated taste cells that showed calcium responses to 10 nM SSP (1–7), a negative control lacking template (−), and whole isolated taste buds used as a positive control (+). Cells 1–3 expressed PLC-β2, but not SNAP-25, and NTPdase II, while cells 4–7 expressed NTPdase II, but not PLC-β2 or SNAP-25.

As an independent confirmation of tachykinin receptor expression in taste cells, RT-PCR was performed on pools of isolated whole taste buds from the circumvallate papillae. The presence of taste bud cDNA was confirmed by detection of PLC-β2, which is an essential component for taste transduction [33], and is not found in surrounding non-taste epithelium [42]. Primers for NK-1R, NK-2R, and NK-3R were validated using cDNA from mouse large intestine (NK-1R, NK-2R) and eye (NK-3R) [43]. NK-1R and NK-2R were expressed taste buds, but not in the surrounding tongue non-taste epithelium. Conversely, NK-3R was not expressed in taste buds but was present in non-taste epithelium (Fig. 7A).

### NK-1Rs are also expressed in Type I (Glial-like) cells

Interestingly, of all identified SSP responders in the PLCβ2-GFP or GAD67-GFP Ca^2+^_-_ imaging experiments, only a subset expressed PLCβ2-GFP (26.6%, or 21/79 SSP-responding cells) or GAD67-GFP (2.3%, 2/86 SSP-responding cells). This indicates that roughly 70% of NK-1R- expressing cells in my isolated taste cell preparation are neither Type II (Receptor)or Type III (Presynaptic) cells, but perhaps the third identified mature taste cell type, so-called Type I (Glial-like) taste cells (Figure 7B). Unfortunately, there is currently no physiological method that can identify Type I (Glial-like) taste cells. As such, to further clarify the identity of NK-1R expressing taste cells, single-cell RT-PCR was performed using RNA isolated from individual taste cells that demonstrated calcium responses to SSP (10 nM). Of 7 isolated SSP-responsive cells, 3 expressed the Type II (Receptor) cell marker PLCβ2, 4 expressed the Type I (Glial-like) cell marker NTPdase II, while none expressed the Type III (Presynaptic) cell marker SNAP-25 (Fig. 7C). Thus, NK-1R appears to be mainly expressed in Type II (Receptor) cells and Type I (Glial-like) taste cells.

An attempt was made to confirm NK-1R expression in taste cells using immunohistochemistry, however the results were inconclusive. Two different commercially available NK-1R-specific antibodies (Cat.# AB5060, Millipore and Cat.# S8305, Sigma-Aldich) positively stained NK-1R knock-out tissue from two independently- created transgenic mouse strains [44], [45], in a manner identical to wild-type tissue, thus calling into question the specificity of the antibodies (images not shown).

### Activation of NK-1R has an additive effect on tastant-induced Ca^2+^-responses in umami Type II (Receptor) cells

**Figure 8:**
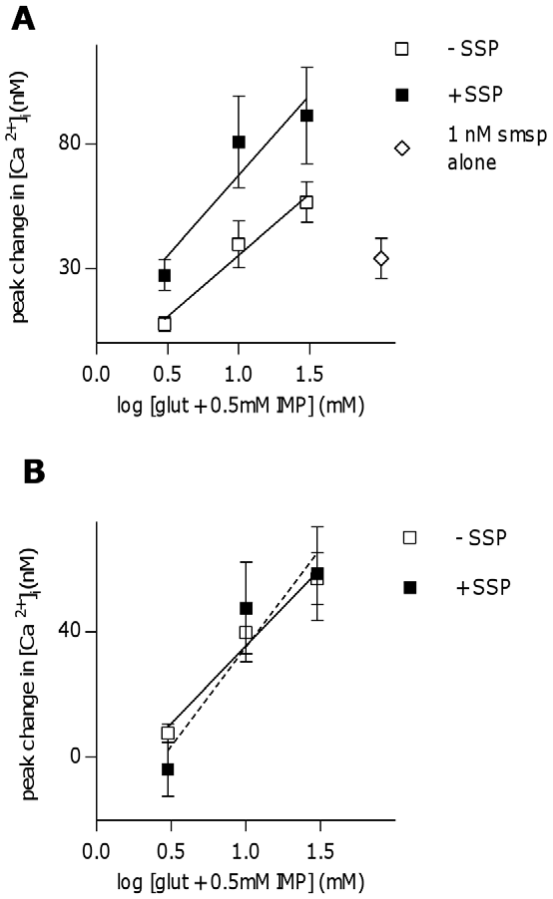
Stimulation of the neurokinin 1 receptor has an additive effect on umami responses in taste cells. **A**, Peak Ca^2+^-responses to 3 concentrations of glutamate (3, 10, 30 mM)+0.5 mM IMP are significantly increased in the presence of 1 nM [Sar^9^,Met(O_2_)^11^]-Substance P (SSP, 1 nM) in SSP-responsive umami taste cells (p = 0.03682, linear regression analysis). Open squares represent averaged responses to glutamate+IMP alone, closed squares represent averaged responses to glutamate+IMP+1 nM SSP, while open diamond represents average response to 1 nM SSP alone. **B**, When peak response to SSP alone is subtracted from responses to glutamate+IMP+SSP in each cell (dotted line), there is no difference in responses as compared to glutamate+IMP alone (solid line).

As NK-1R appears to be expressed in Type II (Receptor) cells, with the majority of these being umami-sensitive, it was next determined if stimulating NK-1R affects taste responses in these cells. Peak calcium responses to three concentrations of glutamate +0.5 mM IMP (3, 10, and 30 mM) were compared in the presence and absence of SSP (1 nM). Responses to glutamate+IMP were significantly larger in the presence of SSP (P<0.05, linear regression analysis) (Fig. 8A). Shown in Fig. 8B, when responses to SSP alone were subtracted, there is no difference between umami responses in the presence or absence of SSP, indicating that the effects were additive, at least at the concentrations of stimuli used in this study.

## Discussion

In this study it has been demonstrated that circumvallate taste buds in mice express NK-1 and NK-2, but not NK-3, tachykinin receptors. In addition, it has been shown that the majority of tachykinin sensitive taste cells appear to be Type I (Glial-like) cells as well as Type II (Receptor) cells that express receptors for the taste modality of umami. The fact that activation of NK-1R excites taste Type II (Receptor) cells and can have an additive effect on the Ca^2+^-responses to taste stimulus suggests that tachykinins such as SP may act as a flavor enhancer. The mechanism of this enhancement is most likely due to concurrent production of IP_3_ through G-coupled protein signaling, as both tachykinins and the taste signaling in Type II (Receptor) cells receptors use this pathway [31]. As SP is found in TRPV1 expressing trigeminal fibers surrounding and penetrating into taste buds [10], [11], it suggests a possible mechanism by which SP-mediated enhancement of taste may occur. One can speculate that consumption of food products containing “spicy” chemicals such as capsaicin, or foods at an increased temperature, can excite TRPV1 on the nociceptive trigeminal fibers, which in turn release SP into the areas in and around taste buds. SP then may concurrently stimulate taste Type II (Receptor) cells along with the taste stimuli at the taste pore, resulting in enhanced taste cell responses. This may explain the paradox of why some people enjoy consuming food containing compounds such as capsaicin, which is normally considered a pain-inducing stimulant. As Type I (Glial-like) taste cells also express NK-1R, SP may also alter salt taste transduction through direct action on Type I (Glial-like) cells, which may be responsible for the transduction of salt taste [46]. Type I (Glial-like) cells may also be responsible for removal of neurotransmitters such as glutamate and ATP, as well as regulation of K^+^ homeostasis in the taste bud [38], thus it would be interesting in future studies to determine if SP has any effect on these functions.

It should be noted that activation of TRPV1 directly on taste cells may affect taste perception, as recent studies have shown the presence of TRPV1 in taste cells [47], [48], and that capsaicin inhibits voltage-gated currents and increased intracellular Ca^2+^ in rat taste cells [49], [50]. However, conflicting studies have shown TRPV1 expression in nerve fibers in and around taste buds, but not in taste cells themselves [51], [52]. In addition, capsaicin altered taste preference to sucrose in TRPV1−/− mice [50], suggesting a possible TRPV1-independent mechanism for capsaicin alteration of taste. Nonetheless, SP may only partly explain the effect of spicy foods on taste, given as capsaicin may directly affect taste cells. It should also be noted that although only rare responses to tachykinins in circumvallate sweet and bitter taste Type II (Receptor) cells were observed, it cannot be ruled out that there is a larger role for tachykinins in bitter, sweet or other taste modalities in other taste bud containing regions of the tongue and mouth, such as the foliate, fungiform, and soft palate.

Unexpectedly, a large number of taste Type II (Receptor) cells that responded to two taste modalities were observed, namely bitter-umami dual responders and sweet-umami dual responders. Sweet or bitter cells that responded to glutamate without enhancement by IMP may be taste cells that express glutamate receptors other than the T1R1-T1R3 umami receptor, as previous studies have shown evidence for this [24], [25], [27], [28], [30], [53]. However, a larger subset of the bitter- or sweet- dual responding cells did in fact show IMP-induced enhancement of glutamate responses, suggesting that these were true umami responses and that these cells do express T1R1-T1R3. This is in contrast with a previous study by Tomchik *et al.*, demonstrating that the large majority of taste Type II (Receptor) cells are narrowly tuned to one taste modality [23]. However, to my knowledge this is the first study to examine overlap between sweet, bitter, and umami responsiveness in isolated individual taste cells, whereas Tomchik *et al.* used a lingual slice preparation, with tastants being applied only at the apical tip of taste buds. The isolated cell prep used in this study may have conceivably exposed receptors on taste cells that are normally hidden or difficult to access from the taste pore *in vivo*, such as receptors that may be present on the basolateral membrane of taste cells. Several early studies on the expression pattern of the T1R1 subunit of the umami receptor using *in situ* hybridization suggested that it was only expressed at low levels in the circumvallate region and did not overlap with sweet (T1R2-T1R3) or bitter (T2R) receptors [54], [55]. However, this contrasted with a later study by one of these groups, in which a more sensitive probe revealed widespread expression of T1R1 in the circumvallate region, as well as a high degree of overlap with T1R2 and moderate overlap with T2Rs, which fits well with the physiological data of this study [40]. In addition, several physiological studies have shown that circumvallate taste buds and cells respond robustly to umami stimuli [23], [24], [56]–[58], and that some Type II (Receptor) cells may be tuned to more than one taste modality [41]. Although beyond the scope of this paper, this phenomenon bears further examination to determine if these dual-responding Type II (Receptor) cells do in fact express two types of taste receptor and play a relevant role in taste *in vivo*.

The proportion of total isolated cells in the dissociated taste bud preparation that responded to tachykinin stimulation (∼9%) seems to be quite low when compared to the proportions of physiologically identified taste cells (in particular umami-responding Type II cells) that responded to tachykinins. A possible explanation for this is the fact that in addition to Type I (Glial-like), Type II (Receptor), and Type III (Presynaptic) taste cells, the dissociated taste bud preparations also contain Type IV Basal cells and immature taste cells [38]. In addition, these preparations also likely contain taste cells that are unresponsive to stimuli due to the fact they are dead or dying, and a small number of non-taste epithelial cells that may have been introduced when taste buds were harvested from the epithelium. Thus, the counts of total fura-2-loaded cells that respond to tachykinins are likely underestimating the true number of tachykinin responsive taste cells. It was also somewhat surprising that only 16% of PLCβ2-GFP cells responded to SSP given the large proportion of umami-responsive cells that also responded to SSP. The most logical explanation for this is that PLCβ2-GFP cells include sweet and bitter Type II (Receptor) cells, along with umami Type II (Receptor) cells. Given that low numbers of sweet and bitter cells responded to SSP (17% and 15%, respectively), one would expect a lower number of PLCβ2-GFP cells (all Type II (Receptor) cells) to respond to SSP as compared to the umami-responding subset of Type II (Receptor) cells. In addition, the number of bitter responsive cells is likely being underestimated when identified with cyclohexamide and denatonium, given that only a subset of bitter cells are activated by these compounds [4].

Taste cells were very sensitive to agonists of NK-1R, responding to SP and SSP at concentrations as low as 3 nM, which is well within the range of known physiological EC_50_ for SP [59]. In contrast, neurokinin A (NKA) only reliably induced Ca^2+^-responses in taste cells at concentrations of 100 nM or higher, with the NK-2R selective agonist [Lys^5^,MeLeu^9^,Nle^10^]-NKA(4–10) showing a similar concentration response range in taste cells. The physiological EC_50_ of NK-2Rs are generally also in the low nanomolar range [60]. This and the fact that both NK-1R and NK-2R antagonists could block NKA responses suggests that NK-2Rs are expressed at much lower levels in taste cells as compared to NK-1Rs. This data indicates that NK-1R is the main active tachykinin receptor in taste cells, with NK-2R likely only being activated by release of very high concentrations of tachykinins. Perhaps the role of NK-2R is to provide additive stimulation to taste cells after stimulation of NK-1Rs has already reached saturation.

Somewhat contradictory to the excitatory effect of tachykinins that was observed on taste cells, there are several physiological and behavioral studies showing an inhibitory effect of capsaicin on various taste qualities [61]–[65]. However, in one of these studies, the initial capsaicin application initially resulted in increased activity in certain units of the NTS [63]. In addition, SP did not modulate CT responses to sweet in the rat [66], suggesting that SP may not be responsible for capsaicin-mediated inhibition of taste responses. Perhaps low concentrations of capsaicin that are near the threshold for somatosensory detection can enhance taste through tachykinin release, while at higher concentrations, other capsaicin-mediated mechanisms may inhibit taste responses. *In vivo* physiological or behavioral experiments will be required to resolve these questions.

There are a wide variety of identified neurotransmitters and signaling molecules that are in and around taste buds and modulate taste bud function, including 5-HT [67]–[69], ATP [70]–[72], norepinephrine [73]–[75], GABA [76]–[78], acetylcholine [79], cholecystokinin [80], neuropeptide Y [81] and glutamate [24], [28], [53]. Substance P and possibly neurokinin A can now also be added to this list. However, an interesting and unique feature of tachykinins is that they appear to primarily affect taste Type II (Receptor) cells of a specific taste modality, namely umami, something that has not been previously demonstrated with any of the other above neurotransmitters and signaling molecules. The full effect of tachykinins in terms of taste perception remains to be determined.
